# Paucity and preferential suppression of transgenes in late replication domains of the *D. melanogaster *genome

**DOI:** 10.1186/1471-2164-11-318

**Published:** 2010-05-21

**Authors:** Vladimir N Babenko, Igor V Makunin, Irina V Brusentsova, Elena S Belyaeva, Daniil A Maksimov, Stepan N Belyakin, Peter Maroy, Lyubov A Vasil'eva, Igor F Zhimulev

**Affiliations:** 1 Department of Molecular and Cellular Biology, Institute of Chemical Biology and Fundamental Medicine SB RAS, Novosibirsk, 630090, Russia; 2 Department of Genetics, University of Szeged, Kozepfasor 52, H-6726, Szeged, Hungary; 3 Institute of Cytology and Genetics SB RAS, Novosibirsk, 630090, Russia

## Abstract

**Background:**

Eukaryotic genomes are organized in extended domains with distinct features intimately linking genome structure, replication pattern and chromatin state. Recently we identified a set of long late replicating euchromatic regions that are underreplicated in salivary gland polytene chromosomes of *D. melanogaster*.

**Results:**

Here we demonstrate that these underreplicated regions (URs) have a low density of *P*-*element *and *piggyBac *insertions compared to the genome average or neighboring regions. In contrast, *Minos*-based transposons show no paucity in URs but have a strong bias to testis-specific genes. We estimated the suppression level in 2,852 stocks carrying a single *P*-*element *by analysis of eye color determined by the mini-*white *marker gene and demonstrate that the proportion of suppressed transgenes in URs is more than three times higher than in the flanking regions or the genomic average. The suppressed transgenes reside in intergenic, genic or promoter regions of the annotated genes. We speculate that the low insertion frequency of *P-elemen*ts and *piggyBac*s in URs partially results from suppression of transgenes that potentially could prevent identification of transgenes due to complete suppression of the marker gene. In a similar manner, the proportion of suppressed transgenes is higher in loci replicating late or very late in Kc cells and these loci have a lower density of *P-elements *and *piggyBac *insertions. In transgenes with two marker genes suppression of mini-*white *gene in eye coincides with suppression of *yellow *gene in bristles.

**Conclusions:**

Our results suggest that the late replication domains have a high inactivation potential apparently linked to the silenced or closed chromatin state in these regions, and that such inactivation potential is largely maintained in different tissues.

## Background

The distribution and suppression of transgenes, and native transposons, can be used as a source of valuable information on genome structure and function. It is known that different retroviruses have different integration bias in mammalian genomes, *e.g*. Human Immunodeficiency Virus has preferences for transcribed units while Murine Leukemia Virus tends to integrate close to active promoters and CpG islands [1]. The distribution of integration sites potentially could be used for identification of active promoters or transcribed units as illustrated by analysis of the transcribed fraction of the human genome using orientation of endogenous transposons [2]. It seems that gene function and expression levels relate to the presence of distinct transposon families in mammalian introns [3]. Long transposon-free regions in mammalian genomes [4] coincide with bivalent chromatin domains associated with key developmental genes in embryonic stem cells [5]. With rare exceptions [6] such transposon-free regions are maintained without apparent conservation of a significant fraction of primary DNA sequence, at least in bony vertebrates, and could be identified only by absence of transposons [7].

It is well established that transgene expression varies in different genomic locations and apparently is linked to the specific chromatin context at the integration site, *e.g*. many transgenes are suppressed in heterochromatic regions [8]. This phenomenon is not limited to the transgenes inserted in pericentric heterochromatin, but is also observed for some transgenes embedded in euchromatic regions of the genome [9]. Therefore variation in transgene expression can be viewed as a special type of position effect ([10] and references therein). On the other hand, only a fraction of transgenes are subject to position effect, so a wide range of domains that are heterogeneous in terms of strength of position effect apparently exist in the genome.

It is most straightforward to relate these peculiar features of transgene expression to the general expression state of the neighboring chromatin. A vast pool of experimental evidence supports this statement. For instance, in a number of model systems reporter genes are inactivated when silencing proteins, such as HP1 or Pc-G proteins, are targeted to their vicinity ([11-15] and references therein). Furthermore, the chromatin state is correlated with the activity of the embedded transgenes [16]. Namely, the chromatin region permissive for transgene expression was shown to be enriched in histone H3K4 methylation and H3 acetylation. In contrast, when transposons were located in regions depleted for these modifications, expression was dramatically suppressed. Consistently, the "open", i.e. active chromatin domains (ridges) in the human genome tended to permit transgene expression, whereas "closed" chromatin domains (anti-ridges) restricted it [17]. Thus, chromatin marks can spread into transgenes and, accordingly, transgene expression can be used as a reporter for the permissiveness of the surrounding chromatin. The distribution of suppressed transgenes provides useful information for analysis of silenced domains [18].

A strong correlation has been reported between transcriptional activity and DNA replication early in S phase in Drosophila and mammals [19,20]. A correlation between the temporal pattern of replication and the density of active transcription for *D. melanogaster *chromosome arm 2L has been described [21], and a positive association between transcription and replication early in S phase has been reported for human chromosome 22 [22]. It seems that the chromatin state also correlates with replication timing, for example the acetylated form of histone H4, H4K16ac, shows a higher correlation with replication timing than with local transcription [23].

In both mammals and fruit flies, early- and late-replication domains are relatively long, varying from 0.1 to 2 Mb [21,22,24,25], and consist of genes with similar replication timing. While it is difficult to predict replication timing based on the expression of a given gene, the position of a gene within a region replicating very late or very early can be a useful predictor of gene expression [26]. The details, however, of the mechanisms linking chromatin state and replication timing remain largely unknown [19].

In Drosophila polytene chromosomes, late replication domains can be seen as transcriptionally silent compacted bands scattered throughout the euchromatic arms. In many ways these domains appear similar to pericentric heterochromatin, and hence were called intercalary heterochromatin back in the 1930s ([27] see references in [28]). It has subsequently been demonstrated that these regions replicate late in S phase [29]. The *SuUR *gene (*Suppressor of Underreplication*) encodes a protein that localizes to regions of intercalary and pericentric heterochromatin and is involved in late completion DNA replication in these regions in endocycling S phase [30,31]. As a consequence, these regions fail to complete replication, and form underreplicated domains, appearing as chromosome breaks (weak points) in polytene chromosome squashes, which serve as cytological markers of late replicating intercalary heterochromatin regions.

In this context we addressed the question of how chromatin replication status correlates with the distribution and suppression of mini-*white- *and *yellow*-marked transposons in *Drosophila melanogaster*. We previously reported the mapping of 52 genomic regions displaying late replication and remaining underreplicated in polytene chromosomes of *D. melanogaster*, due to their failure to complete replication before the end of S-phase [24]. Such Underreplicated Regions (URs hereafter) range from 100 to 600 kb in size and typically encompass 10 to 40 genes. Therefore, URs represent clusters of late replicating genes [24]. We analyzed 2,852 *P*-*element *insertions isolated in genome-wide screens [32,33] for their distribution in relation to URs and for expression levels of their constituent transgenes. Our results demonstrate that both *P-element *and *piggyBac *transposons are depleted in URs compared to adjacent regions or the genome average, and the proportion of suppressed insertions is approximately three times higher in URs than in adjacent flanking regions. In addition we demonstrate that suppression of mini-*white *in eyes and *yellow *in wing cells correlates in transposons carrying two marker genes.

## Results

### *P-element *and *piggyBac *insertions have low density in underreplicated regions

In our work we used 51 known URs in *Drosophila melanogaster*. UR-39DE, containing a histone cluster, was excluded because of inconsistency between the genome assembly and estimated copy numbers of histone genes in the cluster (FlyBase 5.12 annotation compared to [34]). We compared density of insertions from several genome-wide mutagenic projects within 51 known URs and within adjacent fully polytenized genomic regions (flanking regions) (Table 1). These flanking regions used as experimental controls are half of the length of an individual UR on each side, excluding sequences that overlap with other URs. We included in our analysis all insertions in each set with precisely mapped integration sites (mapped to within 10 bp), and we determined the distribution of all unique genomic positions in which insertions were detected (see Methods for details). Such unique sets compensate for multiple insertions into the same location. Six of the seven sets have significantly lower insertion density in URs than in flanking regions or the genome average (Table 1). The only exception is the *Minos*-based transposon *Mi{ET1} *[35] which has essentially no difference in insertion density between URs and flanks (Table 1). Sets of transposons without insulator elements around marker genes such as *P{EP} *or *P{EPgy2} *show the biggest difference, about three times, between URs and flanks, while the transposon with insulators, *P{SUPor-P}*, displays a smaller difference, just 1.9 fold. *piggyBac*-based transposons, *PBac{PB} *and *PBac{RB}*, occur less than half as often in URs than in flanks (Table 1).

**Table 1:** Density of transposons, per Mb, in the underreplicated regions (URs) and flanks.

Collection	URs	Flanks	Genome $	Ratio Flanks/URs	*P *value URs vs Flanks
P{EP} all #	7.3 (118)	23.9 (337)	22.3 (2,649)	3.3	4.0E-42
P{EP} unique	7.1 (114)	20.4 (287)	21.1 (2,509)	2.9	2.7E-32
P{EPgy2} all #	10.7 (172)	34.4 (484)	29.6 (3,526)	3.2	3.3E-59
P{EPgy2} unique	10.6 (171)	34.4 (484)	29.6 (3,515)	3.2	1.8E-59
P{GT1} unique	2.8 (45)	5.8 (81)	4.4 (526)	2.1	5.5E-07
P{SUPor-P} all #	10.9 (175)	21.1 (297)	19.0 (2,259)	1.9	4.0E-19
P{SUPor-P} unique	10.9 (175)	20.7 (292)	18.9 (2,251)	1.9	4.3E-18
Selected set	7.8 (126)	27.0 (381)	24.0 (2,852)	3.5	1.2E-19
PBac{PB} all #	16.6 (268)	37.3 (526)	32.6 (3,883)	2.2	6.1E-42
PBac{PB} unique	14.8 (238)	29.5 (416)	27.4 (3,263)	2.0	1.5E-27
PBac{RB} all #	13.2 (213)	31.3 (441)	28.4 (3,375)	2.4	2.1E-38
PBac{RB} unique	12.8 (206)	30.7 (432)	27.5 (3,271)	2.4	1.9E-38
Mi{ET1} all #	20.3 (327)	21.7 (305)	19.8 (2,351)	1.1	0.2
Mi{ET1} unique	20.3 (327)	21.7 (305)	19.7 (2,348)	1.1	0.2
					
Fraction size, Mb	16.1	14.1	118.9		

# Insertions with the integration sites smaller than 10 bp.

$ Insertions in euchromatic regions of chromosomes X, 2 and 3.

Actual numbers of insertions are shown in brackets.

Estimation of statistical significance of genomic data is a complex problem because many genome characteristics are not normally distributed. Indeed, transgene densities for 51 URs and 94 flank regions apparently do not fit a normal distribution. We tested the significance of the observed paucity of transgenes in URs using the Mann-Whitney U test designed for non-parametric distributions. For this we combined the unique integration sites for *P*-*element*-based transgenes (*P{EP}*, *P{EPgy2} *and *P{GT1}*) and *piggyBac*s (*PBac{PB} *and *PBac{RB}*), estimated transgene density for each UR and flank region, and calculated the probability of obtaining such a distribution by chance using a Mann-Whitney U test on-line calculator [36]. The one-sided *P *values for low transgene density in URs compared to flanks are very low for both sets: 6.9E-10 for the combined set of *P*-*elements *and 1.4E-7 for the combined set of *piggyBac*s. Such low numbers suggest that the observed paucity of *P-elements *and *piggyBac*s is statistically sound.

It is well known that *P-elements *tend to insert close to promoter regions, and this feature potentially could affect the density of transposons in URs. To address these questions we analyzed the distribution of unique genomic integration sites for all transposons sets listed in Table 1 relative to the annotated protein-coding FlyBase genes (Additional file 1 Figure S1). Indeed, *P-elements *are enriched in regions +/- 100 bp from the annotated transcription start sites (TSSs). Next we compared the density of the annotated TSSs of FlyBase genes 5.12 in URs and flanks. The URs contain 1,332 TSSs, and 2,203 are annotated in the flanks. The density of the annotated TSSs in URs is 1.9 folds smaller than in flanks: 82.7 TSSs per Mb compared to 156.4, and this difference is statistically significant (*P *< 1.0E-77, chi-squared test). Nevertheless, the density of *P-elements *in URs is three times lower in URs compared to flanks, and less than half of all *P-elements *are inserted close to TSS (Additional file 1 Figure S1). So, it seems that low TSS density in URs is not the full explanation for the low occurrence of *P-element*-based transposons in URs. The Drosophila gene disruption project aimed for isolation of insertions within genes, and pre-selection of the transgenes before deposition into the database might create some distribution bias. However, *P*-*element*-based transposons *P{EP} *and *P{EPgy2} *and *piggyBac *insertions have a lower density in URs than in flanks in all three analyzed genomic fractions: +/- 100 bp from TSSs, genic and intergenic regions (Additional file 2 Table S1). Hence, the observed difference of the transgene density between URs and flanks unlikely arises from pre-selection of the insertions isolated in the Drosophila gene disruption project.

A recent study demonstrated that transposons are under-represented in testis-specific genes [37]; and many testis-specific genes are located in URs [24]. We investigated whether enrichment of testis-specific genes in URs could explain the paucity of transgenes in these regions. Using the FlyAtlas we selected 1,636 testis-specific genes and 11,056 non-testis-specific genes (see Methods for details). Because *P-elements *tend to insert close to TSSs we analyzed insertions not just in transcribed regions (genes) but also in regions upstream of TSSs. For this purpose we converted genes into loci as follows: we used the most upstream TSS and the most downstream transcription termination site, and added 100 bp upstream of the TSS. In agreement with data reported in [37] all sets of transposons except for *Mi{ET1} *are strongly under-represented in testis-specific loci (Additional file 2 Table S2). However, the difference between insertion densities in testis-specific and other genes is far less dramatic when calculated per length occupied by loci on the genome. Moreover, the insertion density of the *Mi{ET1} *transposon is even two times higher in testis-specific genes if measured per Mb (Additional file 2 Table S2). It is very unexpected because it was reported that *Minos*-based *Mi{ET1} *has integration bias for introns [35] and testis-specific genes in our set are nearly three times shorter than other genes: 2.1 kb *vs *6 kb, hence the intronic fraction would be shorter in these genes.

In agreement with an earlier report [24], the proportion of testis-specific genes is higher in 51 URs compared with the rest of the genome: 30.7% of genes located in URs (331 out of 1,079) are testis-specific by the criteria we used compared to 11.2% (1,305 genes out of 11,613) for the rest of the genome. The total number of genes here is smaller than the number of annotated genes in these regions because not all genes were assayed for transcription. We compared insertion densities within testis-specific genes located in URs and in the rest of the genome (Additional file 2 Table S3). We analyzed insertion density both per 100 loci, and per Mb of genomic DNA occupied by these loci because *P-elements *have a bias to TSSs, and hence comparison per loci would make sense for such insertions while *piggyBac*s apparently have more or less uniform distribution (Additional file 1 Figure S1). Because the numbers of the insertions located in testis-specific loci within URs are small we estimated statistical significance of the observed difference for the combined set of *P-element*-based vectors without insulators, and for the combined set of *piggyBac *insertions. Density of inserts for both combined sets is lower in testis-specific loci located in URs compared to similar loci outside of URs, but this difference is not statistically significant for *P-elements *(Additional file 2 Table S3). The low frequency of *piggyBac *insertions in testis-specific genes in URs suggests that URs have additional restraint(s) to insertion into loci besides the presence of testis-specific genes. In agreement with this assumption, *P-element *insertions into non-testis-specific loci located in URs is less likely compared to similar loci located in the rest of the genome (Additional file 2 Table S3). We would like to point out that both measurements, per locus, and per Mb, show concordant difference.

Our results indicate that the low density of *P-element *and *piggyBac *insertions in URs apparently is not fully explained by low TSS density or enrichment for testis-specific genes in these regions.

### Analysis of 2852 *P-element*-based transposons for suppression

**Figure 1:**
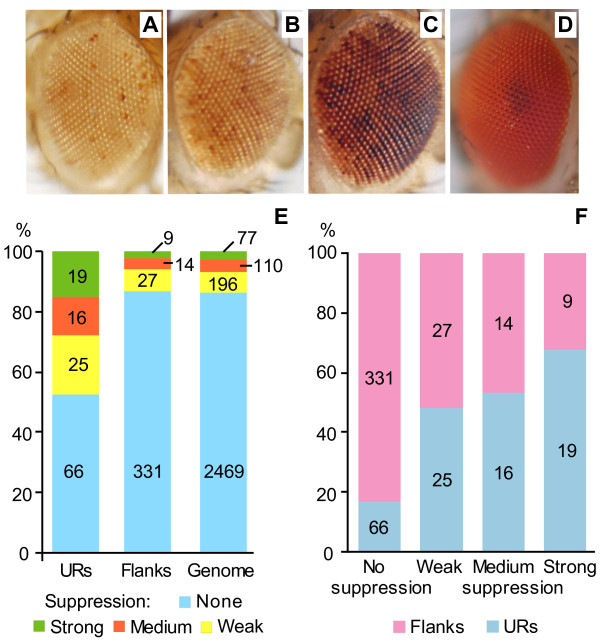
**The underreplicated regions are enriched in suppressed transgenes**. Examples of eye with strong (A), moderate (B) and weak (C) suppression of mini-*white*. (D) Eye color in wild type fly. (E) Distribution of transgenes from Selected set with different suppression of mini-*white *marker gene in URs, the flank regions and the genome. Color coding is explained at the bottom of the graph. The proportion of suppressed transgenes is 3.6 times higher in URs than in flanks (*P *< 7.6E-10). (F) Distribution of transgenes with different level of mini-*white *suppression indicates that URs contain higher proportion of inserts with strong suppression. Probability to have such trend by chance is 3.7E-15.

It is known that many insertions in heterochromatic regions are suppressed. Suppression of marker gene expression makes detection of insertions in such regions nearly impossible. The addition of insulators on both sides of transgene markers results in resistance to suppression and facilitates detection of transposons in heterochromatin [38]. In a similar manner, insulators prevent suppression of marker genes in euchromatin as exemplified in Additional file 3 Figure S2. Considering that the difference in density of *P-elements *with and without insulators between URs and flanks (Table 1) is statistically significant (*e.g*., for unique integration sites of *P{SUPor-P} *and *P{EP} *the *P *value < 0.005; chi[2] = 7.95) we compared suppression of transgenes located in URs and flanks. We estimated mini-*white *expression by eye color in flies with *P{EP} *or *P{EPgy2} *transposons and grouped fly stocks with eye color corresponding to normal mini-*white *expression and with three different levels of variegation (Figure 1). Flies with sectoral type of stable suppression were excluded from the analysis. We used only stocks with homozygous insertions, to control for transgene copy number, and chose only stocks with a single insert per genome. In total, we analyzed 695 stocks with *P{EP} *transposons and 2,157 stocks with *P{EPgy2} *transposons that map to unique positions on chromosomes X, 2 and 3. The distribution of the analyzed subsets in the Drosophila genome is similar to whole sets of *P{EP} *or *P{EPgy2} *transposons (Additional file 1 Figure S1) indicating that the selected transposons have no bias in distribution at least with respect to annotated FlyBase genes. We merged stocks with analyzed eye color into one set, nicknamed Selected set (Additional file 4 Supplemental data 1). Similar to other *P-element *collections, the Selected set is depleted from URs (Table 1) and testis-specific genes (Additional file 2 Table S2). Based on this we conclude that the Selected set has no significant bias in the distribution of insertions in the genome compared to whole *P{EP} *and *P{EPgy2} *sets.

Out of 2,852 insertions in the Selected set, 383 (13.4%) have variegating eye color. If the variegation of eye color resulted from mosaic suppression of the mini-*white *marker gene at the site of integration we would expect enrichment of closely positioned insertions with variegating eye color, namely suppressed insertions would tend to be close to each other. On other hand, if the variegation for each insertion results from some stochastic event, then the suppressed insertions would have a random distribution. For distances ranging from 1 to 5 kb the number of observed pairs in which both transgenes are suppressed is more than double the expected number of such pairs, and the enrichment is higher for shorter distances (Additional file 2 Table S4). This result suggests that suppression of at least some transgenes is linked to chromatin state around the integration site rather than resulting from transposon damage or other artifacts.

### Transposons in URs are preferentially suppressed

In total, 126 inserts analyzed for eye color map to URs, and 60 (47.6%) show some kind of suppression. In stark contrast, out of 381 inserts located in flanks only 50 (13.1%) display evidence for suppression (Figure 1). Thus, the proportion of suppressed transgenes in URs is 3.6 times higher than in flanks (*P *< 7.6E-10, chi[2] = 37.85). Moreover, in URs the proportion of transgenes with stronger suppression is higher than those with weaker suppression (Figure 1). The proportion of the suppressed transgenes in URs is higher in all analyzed genomic fractions but the regions around TSSs and intergenic intervals show the biggest increase compared to flanks or the genome average (Additional file 5 Figure S3).

We investigated whether the high proportion of suppressed transgenes in URs could be linked to enrichment of testis-specific genes in these regions. Only five insertions from the Selected set map to testis-specific loci within URs, and of these, three inserts display suppression of mini-*white*. These numbers are too small to be statistically sound. However, for non-testis-specific genes the proportion of suppressed insertions within loci is 4.2 fold higher in URs than in the rest of the genome: out of 77 inserts from Selected set mapped to non-testis-specific loci in URs, 31 (40.3%) show suppression of mini-*white*, while out of 2,090 inserts mapped to such loci in the rest of the genome, only 201 (9.6%) display mosaic eye color (*P *< 7.5E-12, chi-squared test). These numbers indicate that the higher proportion of suppressed transgenes in URs is not exclusively associated with the presence of testis-specific genes in these regions.

**Figure 2:**
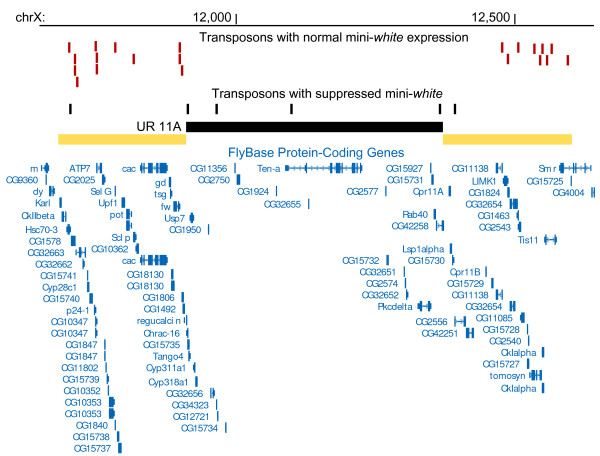
**Example of distribution of the suppressed and active transgenes within and around the underreplicated region in the 11A region of the X-chromosome**. The modified screenshot of USCS genome browser [41] encompassed 1 Mb of genomic DNA (chrX:11,650,001-12,650,000; dm3 genome assembly) is shown. Numbers on top mark the position on the chromosome (in kbs). Red and black bars indicate non-suppressed and suppressed insertions, respectively. Black rectangle corresponds to the underreplicated region and yellow rectangles correspond to the flank regions. Only one annotated isoform of protein-coding FlyBase Genes 5.12 for each gene is shown at the bottom.

In total, 126 inserts from the Selected set are located in 45 URs while 6 URs have no analyzed insertions. Twenty URs contain both suppressed and active insertions (35 and 44 insertions, respectively), while 12 and 13 URs contain either only suppressed or active transgenes, respectively. URs demonstrate various distribution patterns for suppressed and active inserts. Some URs have long clusters of suppressed insertions, *e.g*. four suppressed insertions are present within the underreplicated region located in 11A, UR-11A (Figure 2), and a similar distribution is observed for four suppressed insertions scattered across UR-50C (data not shown). In some cases suppressed insertions cluster in URs, *e.g*. four suppressed insertions map to the distal part of UR-71C. In some URs suppressed and active transgenes are present close to each other, *e.g*. a single suppressed insertion within UR-35E is surrounded by active insertions on both sides. In some cases URs contains clusters of active insertions. Apparently, there is no common distribution pattern for the suppressed insertions in URs, at least for the insertions used in our study.

### Suppression of transgenes correlates in different tissues

**Figure 3:**
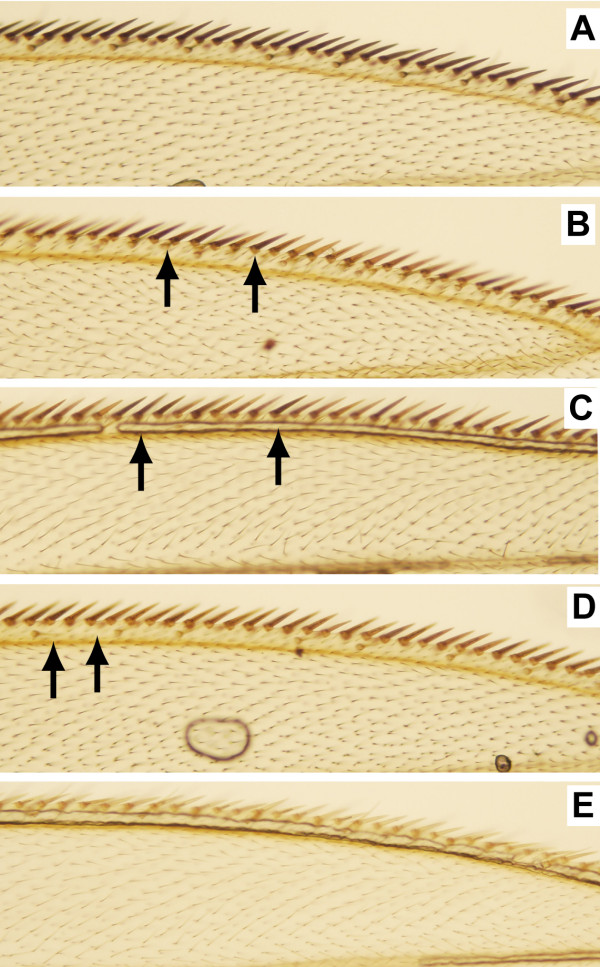
**Suppression of *yellow *gene in bristles**. (A) Wild type wing. Examples of wings with weak (B) and moderate (C) suppression of *yellow *resulting in appearance of both dark and yellow bristles. Some dark bristles are indicated by arrows. (D) Strong suppression of transgene results in very weak staining of nearly all bristles. (E) Wing in *yellow *mutant.

The transposon *P{EPgy2} *contains two marker genes, mini-*white *and *yellow*. Thus, this transposon allows the analysis of suppression in two distinct cell types, eye cells and wing bristle cells, in the same animal (Figure 3). We analyzed 53 EPgy2-bearing stocks with suppressed mini-*white *for suppression of *yellow *(see Methods for details), and only in one stock, *P{EPgy2}EY00386*, suppression of mini-*white *was not accompanied by suppression of *yellow*. In two transgenes, *P{EPgy2}EY02768 *and *P{EPgy2}CG32195*^*EY05483*^, re-examinations have revealed very weak suppression of mini-*white *and no suppression of *yellow*. In *P{EPgy2}CG12797*^*EY11076 *^we detected weak suppression for both mini-*white *and *yellow *while our original analysis suggested no suppression for the same strain. It needs to be pointed out that such observed difference between suppression levels of the marker genes could also depend on amount of product of these genes needed for appearance of normal (wild type) phenotype. Out of 50 transgenes with both mini-*white *and *yellow *suppressed, 27 are located in URs. We conclude that the inactivating potential of regions around of the integration sites tends to be largely the same in both cell types. Very high co-occurrence of suppressed mini-*white *and *yellow *in the same transgene also indicate that suppression is unlikely linked to transposon damage unless we assume that some changes would affect expression of both marker genes.

### *P*-*elements *and *piggyBac*s are depleted from loci replicating late in Kc cells

Next we tested whether low transgene density and the ability to cause transgene suppression are also found in late-replicating regions of another cell type. For this we used comprehensive data on the replication status of nearly all Drosophila genes in Kc cells [23]. We used replication time information for 12,938 FlyBase genes (version 5.12), of which 3,975 are classified as Late Replicated (LR), and 8,963 as Early Replicated (ER). We converted late and early replicated genes into loci by adding 100 bp upstream of the most distant TSS and analyzed insertion densities in these loci. All analyzed transposons sets except for *Mi{ET1} *have low insertion densities in loci replicated late in Kc cells estimated both per loci (Table 2) and per Mb (Table 3). Note that many intergenic transgenes were excluded from this analysis.

**Table 2:** Density of transposons, per 100 loci, in loci replicating late (LR) and early (ER) in Kc cells.

Collection$	in LR loci	in ER loci	Ratio ER/LR	*P *value
P{EP}	6.9 (273)	19.4 (1,738)	2.8	P < 2.2E-56
P{EPgy2}	11.4 (452)	26.0 (2,325)	2.3	P < 1.1E-47
P{GT1}	2.0 (78)	3.1 (274)	1.6	P < 6.1E-4
P{SUPor-P}	7.8 (307)	14.3 (1,281)	1.8	P < 8.1E-21
PBac{PB}	14.3 (566)	18.7 (1,674)	1.3	P < 2.5E-7
PBac{RB}	12.0 (473)	19.2 (1,714)	1.6	P < 8.7E-17
Mi{ET1}	17.9 (710)	9.5 (847)	0.5	P < 1.2E-32
Selected set	7.9 (313)	21.3 (1,908)	2.7	P < 7.5E-58
				
Number of loci	3,956	8,941		

$ Only sets with unique integration sites were used

Actual numbers of insertions are shown in brackets.

**Table 3:** Density of transposons, per Mb, in loci replicating late (LR) and early (ER) in Kc cells.

Collection$	LR loci	ER loci	Ratio ER/LR	*P *value
P{EP}	10.6 (273)	42.3 (1,738)	4.0	1.8E-135
P{EPgy2}	17.5 (452)	56.6 (2,325)	3.2	2.0E-153
P{GT1}	3.0 (78)	6.7 (274)	2.2	5.4E-13
P{SUPor-P}	11.9 (307)	31.2 (1,281)	2.6	5.8E-69
PBac{PB}	22.0 (566)	40.7 (1,674)	1.9	1.9E-50
PBac{RB}	18.4 (473)	41.7 (1,714)	2.3	1.9E-75
Mi{ET1}	27.6 (710)	20.6 (847)	0.7	9.7E-15
Selected set	12.2 (313)	46.4 (1,908)	3.8	5.1E-144
				
Genomic size, Mb	25.8	41.1		

Actual numbers of insertions are shown in brackets.

The LR genes are over-represented in URs (766 LR genes vs 315 ER) compared to the rest of the genome (3,190 LR and 8,626 ER genes; *P *< 7.0E-197 chi[2]= 896.07). This shared characteristic of URs and loci replicating late in Kc cells indicates a significant coincidence of late replicating gene clusters in polytene chromosomes of salivary gland and in embryonic cells. URs are enriched in LR loci but still contain a significant proportion of ER loci, and both ER and LR loci are present outside of URs. Hence we analyzed the distribution of different transgenes in four types of loci: LR in URs, ER in URs, LR outside of URs and ER outside of URs (Additional file 2 Table S5). LR loci have lower *P-element *and *piggyBac *density than ER loci both in URs and in the rest of the genome. Moreover, the difference between densities of *P-elements *and *piggyBac*s in LR and ER loci is bigger in URs (Additional file 2 Table S5). This large difference apparently reflects very low insertion density of *P-elements *and *piggyBac*s in LRs located in URs. The difference between LR loci located in and outside of URs might reflect the degree of late replication of these regions: URs represent very late replicating regions in the salivary gland, while for Kc cells we had not separated late and very late replicating genes. Therefore, we selected 1,132 LR genes replicating very late (replication score equal to or smaller than -2) out of 3,956 LR genes [23] and estimated transposon distribution and suppression in these loci replicating very late in Kc cells. The density of *P{EP} *and *P{EPgy2} *insertions in these loci is approximately half of that in all LR genes, when measured both per loci and per Mb while the difference for *P{SUPor-P} *inserts is smaller. Occurrence of *PBac{PB} *and *PBac{RB} *in very late replicating loci is about 1.5 times lower than in all LR loci, and essentially no difference was observed for *Mi{ET1}*.

*Mi{ET1} *inserts are biased to LR loci (Tables 2 and 3). Above we demonstrated that *Mi{ET1} *inserts are strongly biased to testis-specific genes, and potentially high frequency of *Mi{ET1} *insertions into LR loci could be associated with late replication of a significant proportion of testis-specific genes. Out of 1,636 testis-specific genes in our set, 843 (51.5%) replicate late in Kc cells, while only 30.7% of all analyzed genes (3,956 out of 12,897) replicate late in these cells. We calculated insertion density for late and early replicating testis-specific genes. The information about replication timing in Kc cells is available for 1,628 testis-specific genes. Among these, 843 are labeled as LR, and 785 replicate early. In total, 83 and 70 *Mi{ET1} *insertions were mapped to corresponding loci resulting in very similar insertion density of 44.7 and 43.6 inserts per Mb, respectively, indicating that the high occurrence of *Mi{ET1} *inserts in testis-specific loci does not depend on replication timing of these genes. Density of *Mi{ET1} *inserts into late replicating non-testis-specific genes is 26.4 inserts per Mb, slightly less than *Mi{ET1} *density in all LR loci (27.6 inserts per Mb) but higher than *Mi{ET1} *density in ER loci (20.6 inserts per Mb) (Table 3). This indicates that testis-specific genes contribute significantly to high *Mi{ET1} *density in LR loci but this is not the only factor affecting the observed difference in *Mi{ET1} *integration into LR and ER loci.

### Insertions from Selected set are preferentially suppressed in genes replicating late in Kc cells

**Figure 4:**
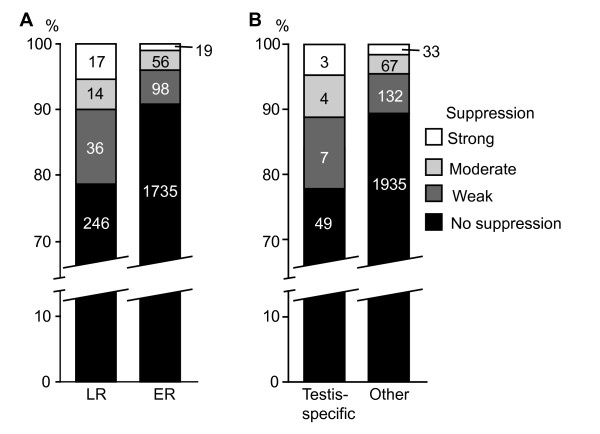
**Preferential suppression of transgenes in testis-specific loci and loci replicating late in Kc cells**. (A) Distribution of the transgens from Selected set in testis-specific loci and all other loci. (B) Distribution of the transgenes in loci replicating late (LR) and early (ER) in Kc cells. Color coding is on the right.

We analyzed the distribution of suppressed insertions from the Selected set mapped to LR and ER loci identified in Kc cells. In total, the proportion of suppressed insertions in LR loci is 2.4 times higher than in ER loci: out of 313 inserts in LR loci 67 (21.4%) were suppressed compared to 9.1% of these in ER loci (173 out of 1,908) (Figure 4A). Next we analyzed suppression in LR and ER loci located in URs and outside URs. The proportion of suppressed inserts in both LR and ER loci located in URs is very high: 46.7% of inserts in LR loci (14 out of 30) and 35.4% of inserts in ER loci (17 out of 48) show some degree of variegation in eye color. Outside of URs, inserts into LR loci have a 2.2 fold higher probability to be suppressed: out of 283 inserts into LR loci outside of URs, 53 (18.7%) are subjected to suppression while within ER loci only 8.4% of the insertions (156 out of 1860) show variegation (*P *< 4.7E-8, chi[2] = 29.8). The results suggest that insertions in URs have a high chance for suppression in the eye regardless of replication time in Kc cells.

It is possible that some types of genes have a high proportion of suppressed inserts, and an obvious candidate would be testis-specific genes, which feature low insertion density. Indeed, out of 63 insertions mapped to testis-specific loci, 14 (22.2%) demonstrate some degree of suppression, which is 2.1 fold higher than the proportion of suppressed insertions in non-testis specific loci: out of 2167 insertions mapped to other genes only 232 (10.7%) show any kind of suppression (*P *< 0.014, chi[2] = 6.0) (Figure 4B). URs contain only three suppressed inserts within testis-specific loci, indicating that testis-specific loci suppress insertions rather independently from URs, and testis-specific genes are not a significant contributor to the high proportion of Selected set suppressed transgenes in URs.

### Distribution of suppressed insertions in the Drosophila genome

We identified Gene Ontology (GO) categories enriched with suppressed insertions from the Selected set. To select genes with suppressed insertions we used gene loci, defined as the region between the most distant transcription start site and the transcription termination site plus 100 bp upstream of TSS. Suppressed insertions are present in 260 loci, and 195 genes have assigned GO categories. We were unable to identify any particular GO category responsible for the suppression of significant proportion of insertion. Nevertheless, some GO categories show enrichment in suppressed elements within corresponding loci, notably GO:0007616 (long-term memory) (Additional file 6 Table S6). However, it needs to be pointed that some GO categories linked to memory such as GO:0007611 (learning and or memory) show enrichment both for suppressed and active transgenes. Moreover, loci with assigned GO categories linked to male gonads such as GO:0007286 (spermatid development) have high proportion of the active insertions (Additional file 6 Table S6) contradicting our observation for higher proportion of suppressed transgenes in testis-specific genes. It seems this contradiction arises from the large difference in size of the datasets used: we analyzed 1,636 testis-specific genes while GO:0007286 (spermatid development) category has been assigned to only 46 genes.

We tested whether transgene suppression depends on conservation of DNA around the integration site by estimating the number of transgenes mapped in phastCons elements [39]. Out of 1151 insertions into phastCons elements, 160 (13.9%) are suppressed which is close to the genome average. Hence, suppression of transposons apparently does not depend on conservation of sequence around the integration site.

It has been suggested that proximity of natural mobile elements, in particular, *1360*, to engineered transgenes on chromosome 4 may cause suppression [40] while subsequent research concluded that the silencing depends on a complex pattern of sequence organization rather than the presence of just one element [18]. While full-size mobile elements are relatively rare in the euchromatic portion of the Drosophila genome and many recent insertions are polymorphic between different Drosophila strains, many sequences resembling natural transposable elements are annotated in RepeatMasker on the UCSC Genome Browser [41]. Among 29 insertions into the annotated repeats that were not included into Selected set (see Methods), 13 (44.8%) demonstrated variegating suppression of mini-*white*. Such a high proportion of suppressed transgenes integrated into the annotated repeats suggests that sequences with similarity to transposable elements may be involved in silencing of transgenes. We analyzed the presence of the annotated repeats (LINEs, LTR and DNA transposons) in the vicinity of the suppressed and active transgenes from the Selected set. In total, the annotated repeat sequences are present within 5 kb from 121 (31.6%) suppressed and 625 (25.3%) active inserts. Annotated LINEs and DNA transposons present more often within 5 kb from the suppressed insertions compared to the active insertions, and the difference is statistically significant (Additional file 2 Table S7). LINEs and DNA transposons also have a higher proportion of bases annotated within 5 kb of integration site of the suppressed insertions (Additional file 2 Table S8). The data suggest that sequences with similarity to transposable elements may play some role in inactivation of some insertions in the euchromatic part of the Drosophila genome. It needs to be pointed out that many sequences annotated as transposable elements in the euchromatic regions of the Drosophila genome are very short and have low similarity score to the canonical elements, and their origin from transposable elements may be in question. Also, the density and proportion of bases annotated as LINEs in URs and flanks are essentially identical, while the density of elements and proportion of bases annotated as DNA transposons in URs are smaller than in flanks, suggesting that a high proportion of suppressed transgenes in URs apparently is not linked to the presence of annotated LINEs or DNA transposons.

We speculate that the low insertion density of *P-elements *and *piggyBac*s in URs and LR loci results in part from complete suppression of transgenes prevents identification of insertions. As a consequence we would expect to see low insertion density in independent sets in regions around the integration sites of suppressed insertions. It seems it is the case for *piggyBac *insertions and *P-elements *without insulators. We analyzed insertion density in 5 kb regions on both sides of the integration sites of suppressed and active transgenes. Because the *P{GT1} *transposon was designed for gene trap screening [42] and hence could have some distribution bias, and *P{SUPor-P} *contains insulators preventing suppression [38], we used an additional set of unique genomic integration sites for the *P{GawB} *transposon [43]. Independent sets of unique integration sites for *P-elements *and *piggyBac*s are biased toward regions adjacent to active transposons while *P-elements *with insulators have an equal chance to be within 5 kb of active or suppressed insertions (Additional file 2 Table S9). It is tempting to speculate that the observed paucity of transposons without insulators around suppressed insertions reflects difficulties in identification of transposons because of suppression.

## Discussion

We have demonstrated that the density of *P*-*element *and *piggyBac *insertions is significantly lower in underreplicated regions of the genome (URs) compared to neighboring regions or the genomic average. In a similar manner, loci replicating late in Kc cells also demonstrate low density of *P*-*elements *and *piggyBac*s compared to early replicating loci. We used independent sets of transposons obtained in genome wide screens [32], so the distribution of the insertions should not be biased to any particular region(s). The paucity of *P-element*-based insertions in URs cannot be explained just by low gene density in these regions because the difference in insertion density is larger than the difference in promoter density. Moreover, *P-elements *are strongly biased towards TSSs but their density is lower around TSSs in URs compared to flanks (Additional file 2 Table S1). It has been reported that *piggyBac *transposons have more or less uniform distribution relative to genes, with some bias to first introns, and the majority of integration events represent a single hit, in contrast to *P-elements *[44]. Despite such distribution, the analyzed sets of *piggyBac*s are under-represented in URs. Target site of *piggyBac *transposons, TTAA, is very short and wide-spread motif [45] and apparently could not be responsible for low occurrence of *PBac{RB} *and *PBac{BP} *in URs.

Testis-specific genes have a low frequency of *P-element *and *piggyBac *insertions (Additional file 2 Table S2) and the proportion of testis-specific genes in URs is higher than in the rest of the genome: 30.7% *vs *11.2% from the total number of genes in these regions. Hence, enrichment of URs with testis-specific genes contributes to some extent to low insertion density in URs. However, non-testis-specific genes in URs have significantly lower density of *P*-*elements *compared to flanks (Additional file 2 Table S3) suggesting other factors (*e.g*. bias of *P-element *transposition events to G2 phase of cell cycle or other factors) apparently contribute to the observed paucity of inserts in these regions.

Distribution of *Mi{ET1} *is opposite to that of *P-element *and *piggyBac *insertions: *Mi{ET1} *inserts are over-represented in both testis-specific and all other loci within URs compared to flanks which is especially pronounced when the insertion density was measured per Mb (Additional file 2 Table S3). The enrichment in URs might be consequence of strong bias of *Mi{ET1} *to testis-specific genes and, to lesser extent to genes replicating late in Kc cells. It was reported that *Mi{ET1} *biased to introns [35]. We calculated the density of available *Mi{ET1} *insertions in exons (24.9 inserts per Mb, based on 724 unique integration sites) and introns (20.7 insertions per Mb, based on 917 unique integration sites). Apparently there is no bias of *Mi{ET1} *transposons towards introns. The nature of such *Mi{ET1} *distribution is not clear. Partially it could be explained by the use of enhanced GFP as a marker gene under the control of a very strong promoter which is significantly less sensitive to surrounding environment than mini-*white *[45], or by pre-selection of the dataset prior to deposition in the FlyBase database.

The *P*-*element *insertions mapped to URs or LR loci have a high proportion of transgenes suppressed in the eye as estimated by variegation of eye color determined by the mini-*white *gene (Figure 1 and 4). We speculate that suppression of transposons in late replication domains may prevent the detection of transgenes during screening, and hence potentially could contribute to the observed low insertion density in these regions similar to pericentric heterochromatin [38]. Indeed, transgenes with insulators, *P{SUPor-P}*, have a smaller difference between URs and flanks (Table 1) or between loci replicating late and early in Kc cells (Tables 2 and 3). In addition, in contrast to *P*-*elements *without insulators, *P{SUPor-P} *insertions occur at the same density near suppressed and active insertions (Additional file 2 Table S9). Besides chromatin inactivation potential, other factors, such as peculiar DNA composition or DNA structure comprising special palindrome sequences [46,47] may contribute to the observed distribution of transposons. Also, successful transgene integration may rely more on the domain chromatin state, rather than on the transcription activity of a particular gene target.

URs have a larger difference in both transposon density (*P*-*elements *and *piggyBac*s) and the proportion of suppressed transgenes than loci replicating late in Kc cells when compared to control regions or ER loci. However, loci replicating very late in Kc cells have transgene density comparable with URs. We estimated the proportion of suppressed transgenes within loci replicating very late in Kc cells. Out of 31 transgenes from the Selected set mapped to very late replicating loci, 16 (43.2%) are suppressed. Thus, loci replicating very late in Kc cells have a very low insertion density and a high proportion of suppressed transgenes. Apparently URs replicating late in salivary gland polytenes and loci replicating very late in Kc cells are enriched with silenced chromatin.

To sum up, we established several facts important for the characterization of late replication domains: i) paucity of *P*-*element *and *piggyBac *insertions in URs and regions replicating late in Kc cells; ii) high proportion of suppressed *P*-*elements *in these regions; iii) significant overlap between URs and loci replicating late in Kc cells; iv) high correlation between suppression of two marker genes present in the same transposon, mini-*white *and *yellow*, in two different organs. These results indicate that late replication domains that manifest as URs in salivary gland polytene chromosomes and appear as densely packed transcriptionally inactive bands largely maintain a closed chromatin state and late replication timing in other cell types without polytene chromosomes. Cytological observations also revealed a remarkable consistency of late replication patterns in different types of cells such as salivary gland or nurse cells in ovaries in Drosophila and Anopheles [48,49]. We do not mean to suggest a complete identity of replication patterns in different tissues. Recent studies demonstrated the plasticity of replication domains both in Drosophila and mammals, namely a change in replication timing in different cell cultures associated with differentiation [23,50]. Such changes in replication timing were reported for 20% of the mouse genome [25] and might be linked to changes in gene expression. To some extent our data demonstrating significant overlap (about 70%) between late replicating loci in salivary gland polytenes and Kc cells, and a high proportion of insertions suppressed in eye cells in these regions, support this point of view on replication domain organization.

It should be pointed out that URs are enriched with testis-specific genes [24] and may also contain other genes expressed in narrow time intervals during development and/or in just a few cells. Activation of just a few genes may have very weak effects on a domain as a whole, and a domain may maintain its closed state and replication status, if the ratio of active and silenced genes has not reached a critical value needed for changes in chromatin state and replication timing of a domain [50,51].

## Conclusion

We demonstrated that *P-elements *and *piggyBac *transgenes are under-represented within late replication domains of the Drosophila genome. Transgenes inserted into late replication domains of the Drosophila genome have a significantly higher chance to be suppressed compared to transgenes into other regions. Such preferential suppression of transgenes occurs in both genic and intergenic regions of late replication domains suggesting that suppression of transgenes is feature of the domains rather than just reflection of other characteristics such as lower gene density within late replication domains.

## Methods

### Sets of transgenes

Coordinates of integration sites for transposon sets used (Table 1) were downloaded from FlyBase [52] as follows: we searched insertion section with transposon name and wildcard symbol and used HitList Conversion Tools to download the coordinates. We kept inserts with integration sites mapped within 10 bp. For unique sets we removed all but one insert at a given genomic position. Only insertions on chromosomes X, 2 and 3 were used (excluding these in heterochromatin).

### Analysis of transposons for suppression

We screened the fly stock collections of *P{EP} *and *P{EPgy2} *transposons available from Bloomington and Szeged Stock Centers. All transgenes were either mini-*white*-marked, or had both mini-*white *and *yellow*. Mini-*white *generally has high level of expression resulting in red/brown eye color. The intronless *yellow *gene confers dark pigmentation to the body and wing cuticle [38]. Only flies with homozygous viable single-copy transgenes that have exact molecular mapping data were analyzed. We also filtered out the insertions displaying no mini-*white *expression, *i.e*. with completely white eyes, insertions with stable pattern of suppression, and the insertions into annotated endogenous transposons longer than 200 bp.

Eye color was visually scored in 10-50 males and females, aged for 3-5 days after eclosion at 22°C. Mini-*white *expression typically appears as uniform coloration of all eye facets ranging from orange to close to wild-type red. Phenotypes with suppressed mini-*white *expression were grouped in 3 classes: *i*). Strong suppression: white or close to white background with or without some darker colored facets (Figure 1A); *ii*). Moderate suppression: light-brown background, with frequent darker colored facets, covering less than half of the eye surface (Figure 1B); *iii*). Weak suppression: eye color is close to wild type, but some facets appear distinctly lighter (Figure 1C). We excluded from the subsequent analysis 48 insertions with stable sectoral suppression of mini-*white *and 29 insertions into annotated transposons of which 13 (44.8%) demonstrated variegating suppression of mini-*white*. The final set contains 2852 insertions of which 383 are suppressed. The information on Selected set is available as Additional file 4 Supplemental_data_1 in bed format suitable for UCSC genome browser with coordinates for dm3 genome assembly (the April 2006 assembly, BDGP Release 5). Score 0 indicates insertions without mini-*white *suppression, score 100, 200 and 300 correspond to insertion with weak, moderate and strong suppression.

Wing bristle color was scored on marginal vein bristles on 10-20 wings from 5 to 10 males and females carrying *P{EPgy2} *transposons. Wild-type *yellow *expression results in grey-brownish bristle color. When *yellow*^+ ^is suppressed, we can find the following: *i*) no or very few grey-colored bristles, with most bristles being *yellow*^- ^(strong suppression); *ii*) both dark- and light-colored bristles are equally prominent on the wing (moderate suppression); *iii*) grey bristles dominate, with only few yellow bristles present (weak suppression) (Figure 3).

### Statistical analysis

We used Pearson chi-square test for 2 × 2, 2 × 3 tables without any corrections. Distribution of insertions per locus was tested as following: number of insertions on each type of loci, e.g. in ER and LR loci was compared to number of corresponding loci. For insertion density (per Mb) the genome total (euchromatic part of chromosomes X, 2 and 3) insertion density was taken as an expected for calculating the expected insertions number for the given region genome span (Mb):

where *O*-observed number of insertions of a given type in a given region; *l*-length in Megabases of a given region; *E*-insertions density of a given type in the region. The *P *value was calculated using CHIDIST function in Excel with d.f. = 1. Statistical significance of the trend on Figure 1F was calculated using contingency tables 2 × 4, d.f. = 4.

The *P *value for distribution of testis-specific and late/early replicating genes in URs and in the rest of the genome was calculated by CHITEST function. Expected values were calculated assuming uniform distribution of the genes (proportional to length of URs and the rest of the genome). The expected promoter density in URs and flanks was calculated assuming uniform distribution of promoters in these regions. For Mann-Whitney U test we used Ivo Dinov's on-line calculator from Statistics Online Computational Resource (SOCR) [36].

### Genomic analysis

Genomic analysis was done on UCSC genome browser web site [41]. Data on replication timing in Kc cells were described in [23]. Testis-specific genes were extracted from the FlyAtlas dataset [53]. Therefore only the genes with reproducible transcription signals (in 2 or more experiments) were used. Genes that showed up-regulation in testis and down-regulation or no expression in all other tissues were regarded as testis-specific. We used FlyBase Genes annotation version 5.12 [52] available on UCSC genome browser web site [41]. Updated coordinates for URs except for region UR-39DE (dm3 assembly) are provided as Additional file 7 Supplemental_data_2. Flank regions were selected as following: for each UR we added half of its size on both sides. Sequences overlapping with other URs were excluded from flanks. For Gene Ontology analysis we used High-Throughput GoMiner [54]. Statistical significance of clustering of suppressed insertions (Additional file 2 Table S4) was estimated by chi-square test, and expected number of suppressed pairs was estimated as following:

where N_exp_-expected number of pairs with both insertions suppressed, N - size of the group (number of pairs separated by no more than 1, 3 or 5 kb), n_sup_-number of suppressed inserts, n_all_-all insertions in the genome.

## Competing interests

The authors declare that they have no competing interests.

## Authors' contributions

ESB and IFZ designed the study, IVB and ESB analyzed the flies, VNB, IVM, DAM, SNB contributed to bioinformatics analysis, PM helped with the fly work, LAV advised on statistics, IVM and ESB wrote the manuscript.

All authors read and approved the final manuscript.

## Supplementary Material

Additional file 1 **Supplemental Figure S1: Distribution of different transposons in the Drosophila genome**. Figure S1. Distribution of different transposons in the Drosophila genome. (A) First column depicts proportion of assembled euchromatic regions of chromosomes X, 2 and 3 occupied by following fraction: 100 bp on both sides from the annotated Transcription Start Sites (TSS) of protein-coding FlyBase Genes 5.12 (+/- 100 bp from TSS), regions between annotated TSS and transcription termination sites except regions occupied by first fraction (Genic-100 bp), and the rest of the genome (Intergenic-100 bp). Other columns show occurrence of different transposons (unique sites) in each genomic fraction. Number of integration sites in each fraction is indicated on the column, and name of transposon vectors are shown below the graph. (B) Distribution of the insertions selected for the analysis of suppression is similar to the distribution of whole sets of unique integration sites of *P{EP} *and *P{EPgy2} *transposons. Click here for file

Additional file 2 **Supplemental Tables 1-5 and 7-9**. Supplemental Tables 1-5 and 7-9. Click here for file

Additional file 3 **Supplemental Figure S2: Effect of the insulators on transgene expression**. Two different transposons are integrated into the same position 2,101,726 on chr2R (Release 5, dm3). (A) *P{EPgy2}EY02768 *contains mini-*white *marker gene. (B) *P{SUPor-P}KG00902 *has mini-*white *gene surrounded by Su(Hw) insulators. The insulators prevent mini-*white *from suppression. Click here for file

Additional file 4 **Localization of transgenes analysed for suppression**. Genomic positions of P{EP} and P{EPgy2} transgenes in the *D. melanogaster *genome, BDGP assembly Release 5 (dm3). Click here for file

Additional file 5 **Supplemental Figure S3: Distribution of suppressed and active transgenes relative to FlyBase protein-coding genes 5.12**. The suppression of transposons was analyzed in three fractions: 100 bp on both sides from the annotated Transcription Start Sites (TSS) of protein-coding FlyBase Genes 5.12 (+/- 100 bp from TSS), regions between annotated TSS and transcription termination sites except regions occupied by first fraction (Genic-100 bp), and the rest of the genome (Intergenic-100 bp). Numbers of the active and suppressed transposons in each fraction are indicated on columns. The proportion of the suppressed transposons in the underreplicated regions (URs) is higher in all fractions compared with the control flank regions or whole genome data but the biggest increase occurs in the regions close to TSS and in the intergenic fraction. Click here for file

Additional file 6 Supplemental Table S6: GO categories of genes enriched with suppressed and active transgenes. Click here for file

Additional file 7 **Localization of 51 underreplicated regions**. Genomic positions of 51 URs analysed in the paper in the *D. melanogaster *genome, BDGP assembly Release 5 (dm3). Click here for file
