# Supplementary figures and images for: Paucity and preferential suppression of transgenes in late replication domains of the D. melanogaster genome
Source: BMC Genomics. 2010 May 21;11:318. doi: 10.1186/1471-2164-11-318 (PMC2887417; doi:10.1186/1471-2164-11-318)

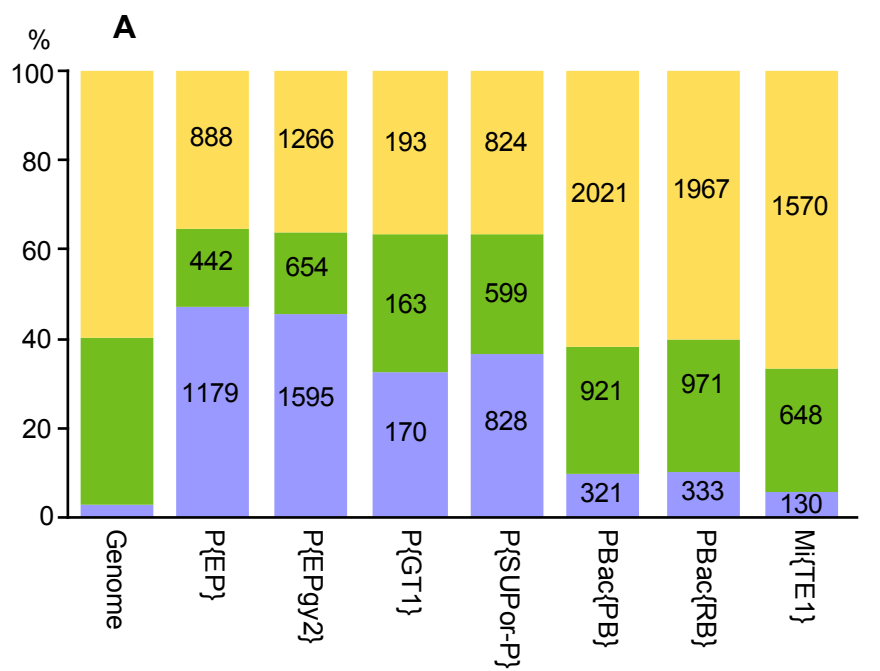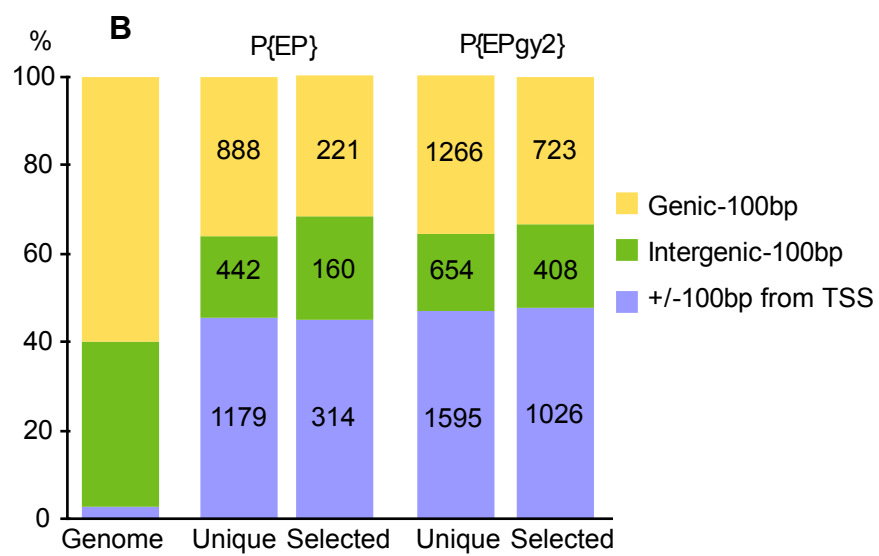

Supplemental\_Figure\_S1

Supplement: Additional file 1 — Supplemental Figure S1: Distribution of different transposons in the Drosophila genome. Figure S1. Distribution of different transposons in the Drosophila genome. (A) First column depicts proportion of assembled euchromatic regions of chromosomes X, 2 and 3 occupied by following fraction: 100 bp on both sides from the annotated Transcription Start Sites (TSS) of protein-coding FlyBase Genes 5.12 (+/- 100 bp from TSS), regions between annotated TSS and transcription termination sites except regions occupied by first fraction (Genic-100 bp), and the rest of the genome (Intergenic-100 bp). Other columns show occurrence of different transposons (unique sites) in each genomic fraction. Number of integration sites in each fraction is indicated on the column, and name of transposon vectors are shown below the graph. (B) Distribution of the insertions selected for the analysis of suppression is similar to the distribution of whole sets of unique integration sites of P{EP} and P{EPgy2} transposons. [file 1471-2164-11-318-S1.PDF]

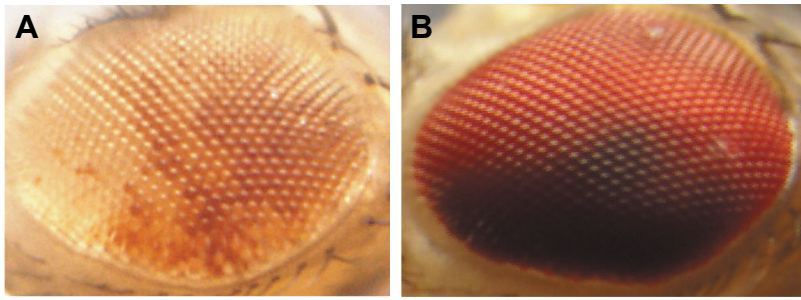

Supplemental\_Figure\_S2

Supplement: Additional file 3 — Supplemental Figure S2: Effect of the insulators on transgene expression. Two different transposons are integrated into the same position 2,101,726 on chr2R (Release 5, dm3). (A) P{EPgy2}EY02768 contains mini-white marker gene. (B) P{SUPor-P}KG00902 has mini-white gene surrounded by Su(Hw) insulators. The insulators prevent mini-white from suppression. [file 1471-2164-11-318-S3.PDF]

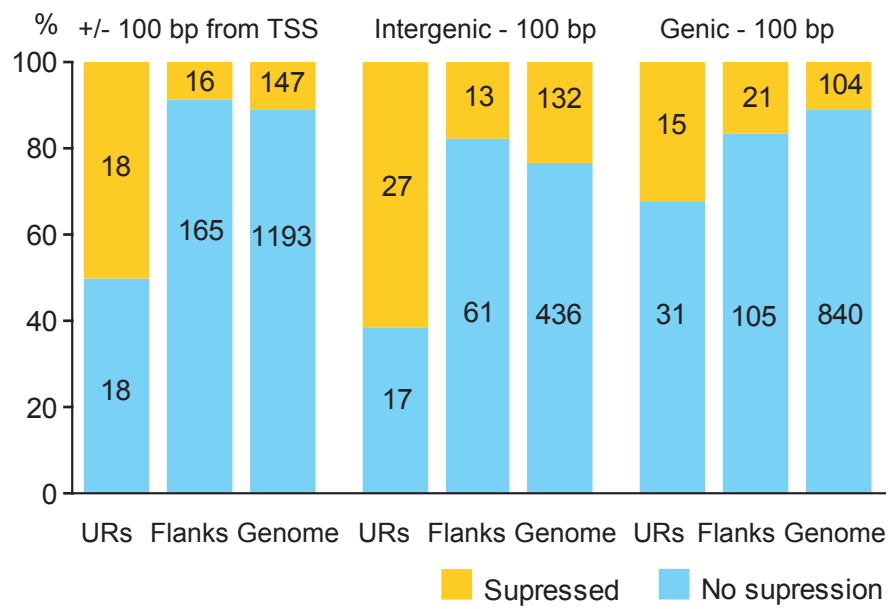

Supplemental\_Figure\_S3

Supplement: Additional file 5 — Supplemental Figure S3: Distribution of suppressed and active transgenes relative to FlyBase protein-coding genes 5.12. The suppression of transposons was analyzed in three fractions: 100 bp on both sides from the annotated Transcription Start Sites (TSS) of protein-coding FlyBase Genes 5.12 (+/- 100 bp from TSS), regions between annotated TSS and transcription termination sites except regions occupied by first fraction (Genic-100 bp), and the rest of the genome (Intergenic-100 bp). Numbers of the active and suppressed transposons in each fraction are indicated on columns. The proportion of the suppressed transposons in the underreplicated regions (URs) is higher in all fractions compared with the control flank regions or whole genome data but the biggest increase occurs in the regions close to TSS and in the intergenic fraction. [file 1471-2164-11-318-S5.PDF]
