# Supplementary material for: Paucity and preferential suppression of transgenes in late replication domains of the D. melanogaster genome
Source: BMC Genomics. 2010 May 21;11:318. doi: 10.1186/1471-2164-11-318 (PMC2887417; doi:10.1186/1471-2164-11-318)
Supplement: Additional file 2 — Supplemental Tables 1-5 and 7-9. Supplemental Tables 1-5 and 7-9. [file 1471-2164-11-318-S2.PDF]

Supplemental Table S1. Density of transposons unique integration sites, per Mb, in different genomic regions based on FlyBase gene annotation 5.12.

Collection	Density in URs	Density in Flanks	Density in Genome	Ratio Flanks /URs	Chi test *P* value
**P{EP}**					
TSS, +/- 100 bp	182.0 (46)	323.6 (136)	374.4 (1,179)	1.8	7.4E-5
Intergenic-100 bp	3.2 (29)	10 (47)	9.9 (442)	3.2	8.1E-11
Genic-100 bp	5.9 (39)	11.6 (104)	12.5 (888)	2.0	1.8E-5
**P{EPgy2}**					
TSS, +/- 100 bp	189.9 (48)	554.4 (233)	506.5 (1,595)	2.9	6.7E-15
Intergenic-100 bp	6.5 (60)	17.7 (83)	14.7 (654)	2.7	1.7E-15
Genic-100 bp	9.5 (63)	18.7 (168)	17.8 (1,266)	2.0	5.4E-8
**P{GT1}**					
TSS, +/- 100 bp	31.6 (8)	61.9 (26)	54 (170)	2.0	0.05
Intergenic-100 bp	2.6 (24)	5.3 (25)	3.7 (163)	2.0	0.0005
Genic-100 bp	2.0 (13)	3.3 (30)	2.7 (193)	1.7	0.06
**P{SUPor-P}**					
TSS, +/- 100 bp	182.0 (46)	278.4 (117)	263 (828)	1.5	0.004
Intergenic-100 bp	8.0 (74)	16.9 (79)	13.5 (599)	2.1	1.2E-10
Genic-100 bp	8.3 (55)	10.7 (96)	11.6 (824)	1.3	0.06
**Selected set**					
TSS, +/- 100 bp	142.4 (36)	430.7 (181)	425.6 (1,340)	3.0	2.7E-12
Intergenic-100 bp	4.8 (44)	15.8 (74)	12.8 (568)	3.3	8.7E-17
Genic-100 bp	6.9 (46)	14 (126)	13.2 (944)	2.0	1.4E-6
**PBac{PB}**					
TSS, +/- 100 bp	83.1 (21)	92.8 (39)	101.9 (321)	1.1	0.6
Intergenic-100 bp	9.0 (83)	21.5 (101)	20.7 (921)	2.4	8.0E-16
Genic-100 bp	20.1 (134)	30.7 (276)	28.4 (2,021)	1.5	1.4E-6
**PBac{RB}**					
TSS, +/- 100 bp	79.1 (20)	99.9 (42)	105.8 (333)	1.3	0.3
Intergenic-100 bp	8.5 (78)	26.4 (124)	21.8 (971)	3.1	1.2E-25
Genic-100 bp	16.2 (108)	29.6 (266)	27.6 (1,967)	1.8	4.1E-10
**Mi{ET1}**					
TSS, +/- 100 bp	63.3 (16)	47.6 (20)	41.3 (130)	0.8	0.3
Intergenic-100 bp	14.8 (136)	16.9 (79)	14.6 (648)	1.1	0.2
Genic-100 bp	26.3 (175)	22.9 (206)	22.0 (1,570)	0.9	0.05

Actual numbers of insertions are shown in brackets.

Chi test was calculated using formula (1) in the Methods section.

Supplemental Table S2. Density of the unique integration sites, per 100 loci, for different transgene sets in testis-specific genes.

Collection	Inserts per 100 loci, testis-specific genes	Inserts per 100 loci, other genes	Ratio	Chi test *P* value
P{EP}	2.2 (36)	17.9 (1,980)	8.1	
P{EPgy2}	4.2 (68)	24.6 (2,720)	5.9	
P{GT1}	0.7 (11)	3.1 (345)	4.6	
P subtotal	7 (115)	45.6 (5,045)	6.5	1E-100
Inserts per Mb, P subtotal	32.3	78.0	2.4	5.2E-22
P{SUPor-P}	3.8 (62)	13.9 (1,532)	3.7	1.2E-25
Inserts per Mb, P{SUPor-P}	17.4	23.7	1.4	0.02
PBac{PB}	4.5 (73)	19.6 (2,168)	4.4	
PBac{RB}	4.1 (67)	19.3 (2,131)	4.7	
PBac subtotal	8.6 (140)	38.9 (4,299)	4.5	6.1E-75
Inserts per Mb, PBac subtotal	39.4	66.5	1.7	6.7E-10
Mi{ET1}	9.4 (154)	12.9 (1,421)	1.4	4.3E-4
Inserts per Mb, Mi{ET1}	43.3	22.0	0.5	7E-16
Selected set	3.9 (63)	19.6 (2,167)	5.1	2.5E-43
Inserts per Mb, Selected set	17.7	33.5	1.9	3.9E-7
Number of loci	1,636	11,056		
Genomic length, Mb	3.6	64.7		

Actual numbers of insertions are shown in brackets.

Locus was defined as a genomic region between most distant annotated transcription termination site and most distant transcription start site plus 100 bp upstream of transcription start site.

P-values for insertion enrichment per locus were calculated using 2x2 contingency tables.
P-values for difference in insertion density were calculated using formula (1) in the Methods section.

Supplemental Table S3. Comparison of transposons integration sites (unique) in testis-specific genes in URs and in the rest of the genome.

	Testis-specific genes insertions per 100 loci (# of insertions)			Other genes insertions per 100 loci (# of insertions)		
Collection	Within URs	Outside URs	Ratio	Within URs	Outside URs	Ratio
P{EP}	0.6 (2)	2.6 (34)	4.3	11.6 (87)	18.4 (1,893)	1.6
P{EPgy2}	3.6 (12)	4.3 (56)	1.2	13.2 (99)	25.4 (2,621)	1.9
P{GT1}	0.6 (2)	0.7 (9)	1.1	2.5 (19)	3.2 (326)	1.2
P subtotal	4.8 (16)	7.6 (99)	1.6	27.4 (205)	47 (4,840)	1.7
Chi test	P<0.1			P<1.6E-11		
Inserts per Mb, P subtotal	24.1	34.2	1.4	37.5	81.8	2.2
Chi test	P<0.19			P<3.5E-29		
P{SUPor-P}	3.9 (13)	3.8 (49)	1.0	11.9 (89)	14 (1,443)	1.2
Inserts per Mb, P{SUP-or}	19.5	17.0	0.9	16.3	24.4	1.5
Chi test	P<0.65			P<2.0E-4		
PBac{PB}	1.5 (5)	5.2 (68)	3.5	19.4 (145)	19.6 (2,023)	1.0
PBac{RB}	1.5 (5)	4.8 (62)	3.2	16.2 (121)	19.5 (2,010)	1.2
PBac subtotal	3 (10)	10 (130)	3.3	35.6 (266)	39.1 (4,033)	1.1
Chi test	P<1.6E-4			P<0.2		
Inserts per Mb, PBac subtotal	15.0	45.0	3.0	48.6	68.1	1.4
Chi test	P<4.5E-4			P<9.0E-8		
Mi{ET1}	10.3 (34)	9.2 (120)	0.9	19.5 (146)	12.4 (1,275)	0.6
Chi test	P<0.6			P<1.4E-6		
Inserts per Mb, Mi{ET1}	51.1	41.5	0.8	26.7	21.5	0.8
Chi test	P<0.28			P<0.01		
Selected set	1.5 (5)	4.4 (58)	2.9	10.3 (77)	20.3 (2,090)	2.0
Chi test	P<0.02			P<1.6E-8		
Inserts per Mb, Selected set	7.5	20.1	2.7	14.1	35.3	2.5
Chi test	P<0.03			P<2.4E-16		
Number of loci	331	1,305		748	10,308	
Length, Mb	0.7	2.9		5.5	59.2	

Locus was defined as a genomic region between most distant annotated transcription termination site and most distant transcription start site plus 100 bp upstream of transcription start site.

P-values for insertion enrichment per locus were calculated using 2x2 contingency tables. *P*-values for difference in insertion density were calculated using formula (1) in the Methods section.

Supplemental Table S4. Expected and observed occurrence of suppressed mini-*white* transgenes in the Drosophila genome.

Distance between transgens	1 kb	3 kb	5 kb
Expected number of suppressed transgenes pairs	8.2	11.6	15.4
Observed number of suppressed transgenes pairs	22	28	34
Ratio observed / expected	2.7	2.4	2.2
Total number of transgene pairs	454	644	855
Chi test *P* value	1.05E-06	1.17E-06	1.73E-06

Chi test was calculated using formula (1) in the Methods section.

Supplemental Table S5. Comparison of transposons unique integration sites (per 100 loci) in loci replicating late (LR) and early (ER) in Kc cells within URs and the rest of the genome.

Collection$	Density in LR within URs	Density in ER within URs	Ratio	Density in LR outside URs	Density in ER outside URs	Ratio
P{EP}	3.4 (26)	19.7 (62)	5.8	7.7 (247)	19.4 (1,677)	2.5
P{EPgy2}	6.4 (49)	17.8 (56)	2.8	12.6 (403)	26.3 (2,271)	2.1
P{GT1}	1.7 (13)	2.2 (7)	1.3	2.0 (65)	3.1 (267)	1.6
P subtotal	11.5 (88)	39.7 (125)	3.5	22.4 (715)	48.9 (4,215)	2.2
Chi test	P<8.8E-17			P<5.2E-68		
Inserts per Mb, P subtotal	19,8	81,1	4.1	33,6	106,6	3.2
Chi test	P<3.1E-28			P<4.5E-200		
P{SUPor-P}	7.3 (56)	14.3 (45)	2.0	7.9 (251)	14.3 (1,237)	1.8
Chi test	P<1.2E-3			P<4.1E-17		
Inserts per Mb, P{SUPor-P}	12.6	29.2	2.3	11.8	31.3	2.7
Chi test	P<1.4E-05			P<1.1E-48		
PBac{PB}	9.7 (74)	24.8 (78)	2.6	15.4 (492)	18.5 (1,596)	1.2
PBac{RB}	6.1 (47)	25.1 (79)	4.1	13.4 (426)	18.9 (1,638)	1.4
Subtotal	15.8 (121)	49.8 (157)	3.2	28.8 (918)	37.5 (3,234)	1.3
Chi test	P<1.4E-26			P<5.7E-10		
Inserts per Mb, PBac subtotal	27.2	101.9	3.8	43.1	81.8	1.9
Chi test	P<7.6E-32			P<5.9E-68		
Mi{ET1}	17.4 (133)	12.4 (39)	0.7	18.1 (577)	9.4 (808)	0.5
Chi test		P<0.08			P<2.9E-30	
Inserts per Mb, Mi{ET1}	29.9	25.3	0.8	27.1	20.4	0.8
Chi test	P<0.36			P<2.1E-07		
Selected set	3.9 (30)	15.2 (48)	3.9	8.9 (283)	21.6 (1,860)	2.4
Chi test	P<2.5E-9			P<4.7E-42		
Inserts per Mb, Selected set	6.7	31.2	4.6	13.3	47.0	3.5
Chi test	P<4.4E-13			P<2.4E-99		
Number of genes	766	315	0.4	3,190	8,626	2.7
Length, Mb	4.5	1.5		21.3	39.6	

Actual numbers of insertions are shown in brackets.
Locus was defined as a genomic region between most distant annotated transcription termination site and most distant transcription start site plus 100 bp upstream of transcription start site.
P-values for insertion enrichment per locus were calculated using 2x2 contingency tables.
P-values for difference in insertion density were calculated using formula (1) in the Methods section.

Supplemental Table S7. Occurrence of annotated repeats within 5 kb from integration sites of Suppressed and Active transgenes from the Selected set.

Have within 5 kb:	Suppressed, % (number)	Active, % (number)	Ratio Suppressed/Active	Chi test *P* value
RepeatMasker	97.7 (374)	97.0 (2,396)	1.0	0.5
LINE, LTR or DNA	31.6 (121)	25.3 (625)	1.2	0.009
LINE	21.4 (82)	11.5 (283)	1.9	5.9E-08
LTR	13.1 (50)	12.6 (310)	1.0	0.8
DNA	8.6 (33)	4.6 (113)	1.9	0.0008
All insertions	383	2,469		

P-values were calculated using 2x2 contingency tables.

Supplemental Table S8. Proportion of bases (%) occupied by annotated repeats within 5 kb from Suppressed and Active transgenes from the Selected set.

Have within 5 kb:	Suppressed	Active	Ratio Suppressed/ Active
RepeatMasker	5.3 (178 / 3,392)	4.1 (704 / 17,301)	1.3
LINE, LTR or DNA	8.7 (95 / 1,083)	7 (326 / 4,688)	1.2
LINE	5.6 (39 / 704)	3.3 (93 / 2,827)	1.7
LTR	7.6 (35 / 463)	7.8 (185 / 2,365)	1.0
DNA	6.9 (20 / 292)	5.9 (49 / 830)	1.2
Regions around all inserts, Mb	3.5	17.8	

Numbers in brackets show kb of repeats / kb around insertions with the annotated repeats.

Supplemental Table S9. Insertion density, per Mb, within +/- 5 kb from integration sites of Suppressed and Active transgenes from the Selected set.

Unique integration sites	Suppressed	Active	Ratio Active/Suppressed	Chi test *P* value
P{GT1}	6.1 (21)	9.8 (174)	1.6	0.023
P{GawB}	60.3 (209)	76.7 (1,362)	1.3	2.9E-4
P{SUPor-P}	53.1 (184)	49.3 (875)	0.9	0.38
PBac{PB}	42.7 (148)	62.2 (1,105)	1.5	2.3E-6
PBac{RB}	47.3 (164)	71.1 (1,262)	1.5	7.5E-8
Mi{TE1}	12.4 (43)	13.5 (239)	1.1	0.023
Regions around inserts, Mb	3.5	17.8		

Actual numbers of insertions are shown in brackets.

Chi test was calculated using formula (1) in the Methods section
